# Urinary tract infection among fistula patients admitted at Hamlin fistula hospital, Addis Ababa, Ethiopia

**DOI:** 10.1186/s12879-017-2265-4

**Published:** 2017-02-16

**Authors:** Matifan Dereje, Yimtubezinesh Woldeamanuel, Daneil Asrat, Fekade Ayenachew

**Affiliations:** 1 Department of medicine, Collages of medicine and health sciences, Ambo University, Ambo, Ethiopia; 2 Department of Microbiology, Immunology & Parasitology, School of Medicine, Addis Ababa University, Addis Ababa, Ethiopia; 3 Addis Ababa Hamlin Fistula Hospital, Addis Ababa, Ethiopia

**Keywords:** UTI, Fistula patients, Hamlin Fistula Hospital, Addis Ababa, Ethiopia

## Abstract

**Background:**

Urinary Tract Infection (UTI) causes a serious health problem and affects millions of people worldwide. Patients with obstetric fistula usually suffer from incontinence of urine and stool, which can predispose them to frequent infections of the urinary tract. Therefore the aim of this study was to determine the etiologic agents, drug resistance pattern of the isolates and associated risk factor for urinary tract infection among fistula patients in Addis Ababa fistula hospital, Ethiopia.

**Methods:**

Across sectional study was conducted from February to May 2015 at Hamlin Fistula Hospital, Addis Ababa, Ethiopia. Socio-demographic characteristics and other UTI related risk factors were collected from study participants using structured questionnaires. The mid-stream urine was collected and cultured on Cysteine lactose electrolyte deficient agar and blood agar. Antimicrobial susceptibility was done by using disc diffusion method and interpreted according to Clinical and Laboratory Standards Institute (CLSI). Data was entered and analyzed by using SPSS version 20.

**Results:**

Out of 210 fistula patients investigated 169(80.5%) of the patient were younger than 25 years. Significant bacteriuria was observed in 122/210(58.1%) and 68(55.7%) of the isolates were from symptomatic cases. E.coli 65(53.7%) were the most common bacterial pathogen isolated followed by Proteus spp. 31(25.4%). Statistical Significant difference was observed with history of previous UTI (*P =* 0.031) and history of catheterization (*P =* 0.001). Gram negative bacteria isolates showed high level of resistance (>50%) to gentamicin and ciprofloxacin, while all gram positive bacteria isolated were showed low level of resistance (20–40%) to most of antibiotic tested.

**Conclusions:**

The overall prevalence of urinary tract infection among fistula patient is 58.1%. This study showed that the predominant pathogen of UTI were E.coli followed by Proteus spp. It also showed that amoxicillin-clavulanic acid was a drug of choice for urinary tract bacterial pathogens.

## Background

Urinary Tract Infections (UTIs) is an infection caused by the presence and growth of pathogens anywhere in the urinary tract including a kidney, ureter, bladder, and urethra. It is one of the most common bacterial infections in women, and 50% to 60% of adult women experience a UTI during their lifetime [1].

Patients with obstetric fistula (OF) can have frequent bladder infections, incontinence of urine and stool. Many of these patients might live with these conditions for several years. This may further predispose them to health related problems like urinary tract infections [2].

Obstetric fistula (or vaginal fistula) is a severe medical condition in which a fistula (hole) develops between the rectum and vagina (recto-vaginal fistula (RVF)) or between the bladder and vagina (vesico-vaginal fistula (VVF)) after severe or failed childbirth, when adequate medical care is not available [3]. Vesico-vaginal and recto-vaginal fistulas are debilitating complications of obstructed labor, which primarily affect women and girls in developing countries [4].

Other causes include poorly performed abortion, sexual abuse and rape, other surgical trauma, gynecological cancers or other related radiotherapy treatments and, perhaps the most important, limited or no access to obstetrical care or emergency services [5].

It has been cited as one of the most dramatic and physically, psychologically, and socially damaging, yet preventable, complications of labor.

The most common symptom of recto-vaginal fistula is passage of bowl contents through the vagina. It may also cause inflammation of vagina, which result in burning, itching and discharge; or inflammation of bladder which cause frequent and sometimes painful urination. There are also some physical complications like damage to the cervix or pelvic bones, neurological conditions, leakage of urine and/or feces into the vagina, urogenital infections, ammonia dermatitis, genital lacerations, kidney infections and amenorrhea [3].

Women with obstetric fistula also face other significant physical and social challenges, including infertility, social isolation and unemployment [6].

In general, obstetric fistula treatment needs prolonged hospitalization and bladder catheterization which may contribute for the development of urinary tract infections in women who have OF.

Although it is difficult to determine precise rates, according to the World Health Organization (WHO) and the United Nations Population Fund Agency (UNFPA), estimated 50,000 to 100,000 women develop obstetric fistulas each year admitted at Hamlin fistula hospital, Addis Ababa, Ethiopia [7].

## Methods

### Study design, area and period

A cross sectional study was conducted from February to May 2015 at Hamlin Fistula Hospital, Addis Ababa, Ethiopia. The hospital is located in Addis Ababa at Lideta subcity

In Ethiopia, it is estimated that 9 000 women annually develop a fistula, where only 1200 of them are treated [8]. However, data on impact of UTI on obstetric fistula patients and distribution and antimicrobial drug susceptibility patterns among urinary pathogen isolated from such patients are scarce. Therefore, the aim of this study was to determine the prevalence of UTI, antimicrobial susceptibility pattern of bacterial isolates and the associated risk factors among obstetric fistula patients.

### Study population

During the period from February to May, a total of 210 fistula patients admitted to the Hamlin Fistula Hospital were screened for significant bacteriuria. The study populations were all fistula patients who were treated during the study period. All consenting fistula patients selected as study participant were included in the study. After obtaining informed consent, study participants were interviewed about their socio-demographic characteristics, presence and duration of their clinical manifestations and information on related risk factors by using a structured questionnaire.

### Culture and identifications

About 10 to 20 ml clean-catch mid-stream urine was collected from fistula patients. The sample was inoculated onto cysteine lactose electrolyte deficient agar (CLED) media and blood agar with calibrated loop of 0.001 ml [9]. The inoculated media was incubated overnight (18–24 h) at 37 °C. After overnight incubation, the bacterial growth on the respective media was observed, and total colony count was done and checked for significant bacteriuria [10].

A significant bacteriuria was considered if urine culture yields ≥10^5^ CFU/mL midstream urine. All positive urine cultures showing significant bacteriuria was sub cultured and further identified by their characteristics appearance on their respective media (colony morphology) and confirmed by the pattern of biochemical reactions (indole production, citrate utilization, motility test, urease test, oxidase test, coagulase and catalase tests) using the standard procedures [10, 11].

### Antimicrobial susceptibility testing

Disk diffusion method was employed for antibiotic susceptibility testing as recommended by CLSI [12]. Mueller-Hinton agar (Oxoid) was used for susceptibility testing. Antibiotics discs (Oxoid Ltd) used were: Ceftriaxone (CRO) (30 μg), Chloramphenicol (C) (30 μg), Gentamicin (CN) (10 μg), Ciprofloxacin (CIP) (10 μg),) Nitrofurantion (F) (300 μg), and amoxicillin-- clavulanic acid (AMC) (30 μg).

Briefly pure bacterial culture was transferred into a tube containing 5 ml sterile normal saline (0.85% NaCl) and mixed gently until it forms a homogenous suspension. The turbidity of the suspension was adjusted to the optical density of McFarland 0.5. A standard inoculum adjusted to 0.5 McFarland was swabbed on to Muller- Hinton agar (Oxoid) and antibiotic discs were dispensed after drying the plate for 3–5 min and incubated at 37 °C for 24 h. Diameter of the zone of inhibition around the disc was measured to the nearest millimeter using a metal caliper and the isolate was classified as sensitive, intermediate and resistant according to [12].

### Quality control

*E. coli* (ATCC 25922), *S. aureus* (ATCC25923) and *P. aeruginosa* (ATTC 27853) were used as reference strains for culture and sensitivity testing throughout the study [12].

### Statistical analysis

Data was entered and analyzed using SPSS version 20 software. Odds ratio was used to screen the possible potential risk factors and to compare the proportion of bacterial isolates with patients’ demographic information and comparison of antimicrobial resistances. *P*-value <0.05 was considered statistically significant.

## Results

The data collected in this study consisted of 210 fistula patients admitted to Hamlin Fistula Hospital for surgical repair. All were investigated for presence or absence of urinary bacterial pathogen during the study period between February and May, 2015. The age range of study participants was 12 to 42 years (mean age of 21 years). Majority of the study participants 118 (56.2%) were in the age group of 21–25 years and 196(93.3%) were from rural settings. Overall 134(63.8%) of the study participant had less than 500 ETB personal monthly incomes. A high proportion 77(36.7%) of the study participants are divorced and 99 (47.1%) had a previous history of catheterizations (Table 1).

**Table 1:** Socio demographic characteristics of 210 Fistula patients investigated for UTIs at Hamlin Fistula hospital, Addis Ababa, Ethiopia

Variables	Frequency	Percent (%)
Age (years)		
10–15	2	1.0
16–20	49	23.3
21–25	118	56.2
26–30	25	11.9
31–40	9	4.3
36–40	6	2.9
>40	1	0.5
Residence		
Rural	196	93.3
Urban	14	6.7
Educational status		
Illiterate	163	77.6
Primary school	44	21.0
Above primary school	3	1.4
Occupations		
Merchant	34	16.2
Farmer	62	29.5
Student	17	8.1
House Wife	67	31.9
Daily Laborer	23	11.0
Others	7	3.3
Marital status		
Single	72	34.3
Married	61	29.0
Divorced	77	36.7
Personal income (ETB)		
Less than 500	134	63.8
500–1000	51	24.3
1001–1500	17	8.1
Above 1500	8	3.8
Previous history of catheterization		
Yes	99	47.1
No	111	52.9
Previous history of UTI		
Yes	85	40.5
No	125	59.5

*ETB* Ethiopian birr

Significant bacteriuria was observed in 122 (58.1%) of 210 fistula patients screened for urinary tract infections (Table 3). The overall prevalence of bacterial isolates of the current study was 58.1% and 68(55.7%) of the isolates were from symptomatic cases (Table 2).

**Table 2:** Significant bacteriuria among symptomatic and asymptomatic fistula patient’s investigated for UTIs in Hamlin Fistula Hospital, Addis Ababa, Ethiopia

Fistula patients with UTI	Significant bacteriuria		Total	OR(95% CI)	*P* value
	Yes	No			
Symptomatic No. (%)	68(58.1)	49(41.9)	117(55.7)	1.002(0.577–1.74)	0.994
Asymptomatic No. (%)	54(58.1)	39(41.9)	93(44.3)		
Total No. (%)	122(58.1)	88(41.9)	210(100)		

Of the total of 117 (55.7%) symptomatic cases 68 (58.1%) of them were positive for significant bacteriuria while from the total of 93(44.3%) asymptomatic cases 54(58.1%) were positive for significant bacteriuria. The odds of developing UTI for both symptomatic and asymptomatic patients are the same, OR 95% CI (1002(0.577–1.74), which indicate there is no association between clinical sign and significant bacteria isolates. In general no statistically significant differences were observed in the isolation frequency of each pathogen in the two groups (*p >* 0.05) as shown in Table 2.

Of the 122 isolates only 5(4.1%) of them were gram positive bacteria while nearly all 117 (95.9%) were gram negative bacteria. *E. coli* 65(53.7%) were the commonest bacterial pathogen isolated and followed by *Proteus* spp.31 (25.4%). *Klebsiella spp.* and *Pseudomonas spp.* accounted for 14(11.5%) and 4(3.27%) respectively. Others found in small number included *Serratia spp.*3 (2.46%), *Coagulase negative Staphylococcus* 3 (2.46%) and *S. aureus 2(1.64%). Serretia spp., coagulase negative staphylococcus and S. aureus* were only isolated from symptomatic fistula patients (Table 3).

**Table 3:** Frequency and types of bacterial species isolated from asymptomatic and symptomatic UTI among fistula patients at Hamlin Fistula Hospital, Addis Ababa, Ethiopia

Bacteria species isolated	Symptomatic UTI No. (%)	Asymptomatic UTI No. (%)	Total No. (%)
*E. coli*	31(45.5)	34(62.9)	65(53.3)
*Klebsiella spp.*	9(13.2)	5(9.2)	14(11.5)
*Pseudomonas spp.*	2(2.9)	2(3.7)	4(3.3)
*Proteus spp.*	18(26.4)	13(24.7)	31(25.4)
*Serratia spp.*	3(4.4)	0(0.0)	3(2.45)
*CONS*	3(4.4)	0(0.0)	3(2.45)
*S.aureus*	2(2.9)	0(0.0)	0(0.0)
Total	68(55.7)	54(44.3)	122(100)

*CONS* cougulase negative staphylococcus

### Risk factors associated with urinary tract infections

Significant bacteriuria was strongly associated with history of previous UTI and history of catheterizations (*p <* 0.05) as shown in Table 4. Statistical significance difference was observed in relation to previous history of catheterization and UTI with OR (95%CI) 2.739(1.547, 4.849), *P* value = 0.001 and OR (95%CI) 1.879(1.060, 3.331), *P* value = 0.031 respectively.

**Table 4:** Risk factors associated with significant bacteria isolated from obstetric fistula patients at Hamlin fistula hospital, Addis Ababa, Ethiopia

Variables	Significant bacteria isolated		OR(95%CI), *P*-value
	Yes N. (%)	No N. (%)	
Previous history of catheterization			
yes	70(57.4)	29(33.3)	2.739(1.547, 4.849),0.001
No	52(42.6)	59(67.7)	
Previous history of UTI			
yes	57(46.7)	28(31.8)	1.879(1.060, 3.331), 0.031
No	65(53.3)	60(68.2)	

The average duration of hospital stay among fistula patient screened for UTI was 30 days with range of 1 to 60 days. From admitted patient 38(18.1%) was screened for UTI within two days of admission while 22(18%) of them were with significant bacterial isolates. Of the total 210 study participants 122(58.1%) study participants were diagnosed with significant bacteriuria. The majority these patients 100/122 (82%) stayed in hospital for more than 3 days. There is no statistical significance difference observed in relation to duration of hospital stay with the OR (95%CI) (0.990(0.486, 2.017) and *P* value > 0.05. Educational status, marital status, occupation and other independent variables used were not show statistical significance difference with *P* value >0.05 as shown in Table 5.

**Table 5:** Significant bacteriuria in relation to socio-demographic characteristics of obstetric fistula patients at Hamlin Fistula Hospital, Addis Ababa, Ethiopia

Variables	Significant bacteriuria		Total No. (%)	*P*-value
	Yes No. (%)	No No. (%)		
Age (years)				
10–15	2(100)	0(00)	2(1.0)	
16–20	24(40.7)	25(59.3)	49(23.3)	
21–25	72(61)	46(39)	118(56.2)	0.765
26–30	15(60)	10(40)	25(11.9)	
31–35	6(66.6)	3(33.3)	9(4.3)	
36–40	2(33.3)	4(66.6)	6(2.9)	
Above 40	1(100)	0(00)	1(.5)	
Marital status				
Single	40(55.5)	32(44.5)	72(34.3)	0.856
Married	38(62.9)	23(37.1)	61(29.0)	
Divorced	44(57.1)	33(42.9)	77(36.7)	
Educational status				
Illiterate	97(59.5)	66(40.5)	163(77.6)	0.534
Primary School	23(52.7)	21(46.3)	44(21.0)	
Greater	1(33.3)	2(66.6)	3(1.4)	
Occupations				
Merchant	17(50)	17(50)	34(16.2)	0.52
Farmer	33(53.2)	29(46.8)	61(29.5)	
Student	7(41.2)	10(58.8)	17(8.1)	
House Wife	45(67.1)	22(32.9)	77(31.9)	
Daily Laborer	16(69.6)	7(30.4)	23(11.0)	
Others	4(57.1)	3(42.9)	7(3.3)	
Personal income(ETB)				
Less 500	78(58.2)	56(41.8)	134(63.8)	0.955
500–1000	29(56.8)	22(53.2)	51(24.3)	
1001–1500	11(64.7)	6(35.3)	17(8.1)	
Above 1500	4(50)	4(50)	8(3.8)	
Residence				
Rural	112(91.8)	84(95.4)	196(93.3)	0.31
Urban	10(8.2)	4(4.6)	14(6.7)	
Duration of hospital stay				
Less than 3 days	22(18)	16(18.2)	38(18.1)	0.978
Equal or above 3 days	100(82)	72(81.8)	172(81.9)	

*ETB* Ethiopian birr

### Antimicrobial susceptibility testing

#### Gram negative bacteria

The resistance pattern of gram negative bacteria (*n =* 117) against 6 antimicrobial agents are shown in Table 6. Gram negative bacteria isolates showed low level of resistance to most of antimicrobial tested, 21.4% to amoxicillin -clavulanic acid and ceftriaxone and 33.3% to both nitrofurantoin and chloramphenicol. Intermediate level of resistance (3.4– 7.7%) was observed to most of antimicrobial tested except amoxicillin -clavulanic acid. High level of resistance (>50%) was observed to gentamicin and ciprofloxacin. Among the isolates *Klebsiella spp.*shows low rate of resistance to ceftriaxone (14.3%) and nitrofurantoin (7.5%), while the same rate of intermediate resistance level (7.5%) to gentamicin, nitrofurantoin and ceftriaxone were observed. The isolate also shows high rate of resistance to ciprofloxacin (78.6%).

**Table 6:** Antimicrobial resistance pattern of gram negative bacteria isolated from fistula patient at Hamlin Fistula Hospital, Addis Ababa, Ethiopia

Bacterial isolates		Antimicrobial Tested					
		AMC	CIP	CN	F	C	CRO
*E. coli* (*n =* 65)	S	51(78.4)	28(43.1)	25(48.5)	36(55.4)	41(63.1)	45(69.2)
	I	0(0.0)	0(0.0)	5(7.7)	3(4.6)	3(4.6)	4(6.1)
	R	14(21.6)	37(56.9)	35(53.8)	26(40)	21(32.2)	16(24.6)
*Klebsiella spp.* (*n =* 14)	S	14(100)	3(21.4)	6(42.8)	12(85.7)	10(71.4)	11(78.6)
	I	0(0.0)	0(0.0)	1(7.1)	1(7.5)	0(0.0)	1(7.5)
	R	0(0.0)	11(78.6)	7(50)	1(7.5)	4(28.6)	2(14.3)
*Pseudomonas spp.* (*n =* 4)	S	1(20)	3(80)	4(100)	1(20)	3(80)	4(100)
	I	0(0.0)	0(0.0)	0(0.0)	0(0.0)	0(0.0)	0(0.0)
	R	3(80)	1(20)	0(0.0)	3(80)	1(20)	0(0.0)
*Proteus spp.* (*n =* 31)	S	25(80.6)	13(41.9)	12(48.7)	20(64.5)	17(54.8)	21(67.7)
	I	0(0.0)	4(12.9)	0(0.0)	2(6.5)	1(3.2)	4(12.9)
	R	6(19.4)	14(45.9)	19(61.3)	9(29)	13(41.9)	6(19.4)
*Serratia spp.* (*n =* 3)	S	1(33.3)	0(0.0)	1(33.3)	3(100)	3(100)	2(66.6)
	I	0(0.0)	1(33.3)	0(0.0)	0(0.0)	0(0.0)	0(0.0)
	R	2(66.6)	2(66.6)	2(66.6)	0(0.0)	0(0.0)	1(33.3)
Total (*n =* 117)	S	92(78.6)	47(40.2)	48(41)	72(61.5)	74(63.2)	83(70.9)
	I	0(0.0)	5(4.3)	6(5.1)	6(5.1)	4(3.4)	9(7.7)
	R	25(21.4)	65(55.5)	63(53.8)	39(33.3)	39(33.3)	25(21.4)

*CRO* Ceftriaxone, *C* Chloramphenicol, *CN* Gentamicin, *F* Nitrofurantoin, *CIP* Ciprofloxacin, *AMC* Amoxicillin- clavulanic acid

*Pseudomonas spp.* shows low rate of resistance (20%) to both ciprofloxacin and chloramphenicol and high rate of resistance (80%) to nitrofurantoin, while it was not showed any resistance level to both gentamicin and ceftriaxone.

*E.coli* was the commonest bacterial pathogen isolated which showed low resistance rate (21.6%, 24.6%, and 32.2%) to amoxicillin- clavulanic acid, ceftriaxone and chloramphenicol, respectively. The isolates also shows high rate of resistance to ciprofloxacin (56.9%) and gentamicin(53.8%). *Proteus spp.*and *Serratia spp.* were also other bacterial isolates which showed the high level of resistance to gentamicin (61.3% and 66.6%) respectively, while they were showed low resistance rate to ceftriaxone (19.4%, 33.3%).

### Gram positive bacteria

The resistance pattern of gram positive bacteria (*n =* 5) against 6 antimicrobial agents are shown in Table 7. All gram positive bacteria isolated were 100% sensitive to Amoxicillin-clavulanic acid and ciprofloxacin. Low level of resistance (20–40%) was observed to all the rest of antibiotic tested.

**Table 7:** Antimicrobial resistance pattern of gram positive bacteria isolated from fistula patients at Hamlin Fistula Hospital, Addis Ababa, Ethiopia

Bacterial isolates		Antimicrobial tested					
		AMC	CIP	CN	F	C	CRO
CONs (*n =* 3)	S	3(100)	3(100)	1(33.3)	2(66.6)	2(66.6)	2(66.6)
	I	0(0.0)	0(0.0)	0(0.0)	0(0.0)	0(0.0)	0(0.0)
	R	0(0.0)	0(0.0)	2(66.6)	1(33.3)	1(33.3)	1(33.3)
*S.aureus* (*n =* 2)	S	2(100)	2(100)	2(100)	2(100)	2(100)	2(100)
	I	0(0.0)	0(0.0)	0(0.0)	0(0.0)	0(0.0)	0(0.0)
	R	0(0.0)	0(0.0)	0(0.0)	0(0.0)	0(0.0)	0(0.0)
Total (*n =* 5)	S	5(100)	5(100)	3(60)	4(80)	4(80)	4(80)
	I	0(0.0)	0(0.0)	0(0.0)	0(0.0)	0(0.0)	0(0.0)
	R	0(0.0)	0(0.0)	2(40)	1(20)	1(20)	1(20)

## Discussion

A variety of enteropathogenic bacteria are known to cause UTI worldwide. UTIs are among the most common bacterial infections in humans, both in the community and hospital settings. It is a serious health problem affecting millions of people each year and is the leading cause of gram-negative bacteremia [13]. Patients with obstetric fistula (OF) can have frequent bladder infections, incontinence of urine and stool. Many of these patients might live with these conditions for several years. This may further predispose them to health related problems like urinary tract infections [2]. However, there is a lack of concrete evidences that show the magnitude of UTI and antimicrobial sensitivity patterns in obstetric fistula patients throughout the world and it is difficult to compare all the current findings with previous reports.

This study finding showed that low socioeconomic status was one of the factors that were not significantly associated with increased UTI (*P* value = 0.955) as indicated in Table 5. This report was the same with other report from Thailand which report insignificant association between UTI and socio economic status [14]. Another study on pregnant women in North West Ethiopia showed that pregnant women who had monthly income of less than 500 Ethiopian birr have (18.9%) more likely to have bacteriuria than those who had high socioeconomic income level [15]. Also other study in Egypt on UTI showed the presence of association between low income level and UTI [16]. This could be due to the relation of low socioeconomic status with nutrition and immunity especially in fistula patients.

This study finding also showed that educational status was one of the factors that was not significantly associated with increased UTI (*P* value =0.534) as indicated in Table 5. The frequency of UTI (59.5%) was higher among illiterate fistula patients when compared with others. This study was the same as other studies which indicate absence of association between level of education and UTI among pregnant women in Pakistan [17] and in Tanzania [18].

In general, the overall prevalence of UTI in the present study was 58.1%, which is almost similar with other report from India (60%) [19]. But the present finding of UTI was lower than other studies from Libya (65.7%*)* [20] and Nigeria (76.1%) [21].

Lower report was also reported from other African country Tanzania [18] and Nigeria (47.5%) [22]. The prevalence of present study was also higher than other reports from Ethiopia; North West Ethiopia [23]; (52.8%) [24], Addis Ababa [25] and Dessie [26].

In this study, the most commonly isolated organisms were *Escherichia coli (*53.7%), *Proteus* spp.(25.4%) and, *Klebsiella spp.*(11.5%). Similar isolate with different frequency was found on study done in Libya, *Escherichia coli (*33.98%), *Proteus* spp. (21.48%) and *Klebsiella pneumoniae(*10.3%) [20].

According to previous study done in West Ethiopia *Citrobacter* (24.5%) [24] was the most dominant bacterial isolated while in this study *Citrobacter* was not isolated and *Escherichia coli (53.7%)* was the most dominant isolated similar to other many studies *(39%, 44.9%, 31.7* %) respectively [19, 27, 28].

In the present study, the prevalence of symptomatic and asymptomatic bacteriuria were the same (58.1%), with the OR (95%CI) of 1.002(0.577-1.74) and *P* value of 0.978, which indicate there is no statistically significant differences observed in the isolation frequency of each pathogen in the two groups (*p >* 0.05) (Table 5). This study was the same as previous study done in North West Ethiopia where no statistically significant differences were observed [28]*.*

The finding of this study also revealed that past history of UTI had strong association with UTI with the OR (95%CI) 1.879 (1.060, 3.331) and *P* value = 0.031 as indicated in Table 4. Similar finding were reported from North West Ethiopia [15, 28]. Another study in Tanzania also reported that past history of UTI was a risk factor for UTI during pregnancy [18]. But absence of association was reported from Thailand [14].

This finding also revealed that history of catheterization had strong association with UTI with the OR (95%CI) 2.739(1.547, 4.849) and *P* value = 0.001, which is almost similar with other reports where catheterization is the most important risk factor for the development of catheter associated bacteriuria [29].

In this report, gram negative bacteria isolated showed high level of resistance to ciprofloxacin and gentamicin (>50%) and intermediate level of resistance (3.4– 7.7%) was observed to most of antimicrobial tested except amoxicillin-clavulanic acid.

This is in contrast to previous study done in Ethiopia, where gentamicin considered as appropriate antimicrobial for empirical treatment of urinary tract infections [26]. According to percent study all gram negative bacteria isolate were sensitive (61.5%–78.6%) to amoxicillin-clavulanic acid, nitrofurantoin, ceftriaxone and chloramphenicol. The same result were also reported from other previous study in Ethiopia were amoxicillin–clavulanic acid was appropriate drug of choice for UTI [30]. Low level of resistance (20–40%) was observed to all gram positive isolates against ceftriaxone, chloramphenicol, gentamicin and nitrofurantoin. This study also reveals 100% sensitivity to Amoxicillin-clavulanic acid and ciprofloxacin for all gram positive bacteria isolates. The same result was reported from other previous Ethiopian studies where low level of resistance (8.6%–34.3) was reported with the same antibiotic tested with the current studies [28].

In conclusion overall prevalence of urinary tract infection among fistula patient was 58.1%. The prevalence of significant bacteriuria among both asymptomatic and symptomatic fistula patients was almost the same. This study showed that the predominant pathogen of UTI were *E.coli*. Significant bacteriuria was significantly associated with history of previous UTI and history of catheterization. This study also showed that amoxicillin-clavulanic acid was a drug of choice for both gram negative and gram positive bacteria while ciprofloxacin and ceftriaxone were found effective against gram negative and positive bacteria isolated respectively.

## Conclusions

The overall prevalence of urinary tract infection among fistula patients was 58.1%. Significant bacteriuria was significantly associated with history of previous UTI and history of catheterization. This study also showed that the predominant pathogen of UTI were *E.coli* and agumantin and ciprofloxacin were the drug of choice for gram positive bacteria while CAF and ceftraxone were found effective against gram negative bacteria isolated.

## Abbreviations

CLED: Cysteine lactose electrolyte deficient agar
CLSI: Clinical and Laboratory Standards Institute
RVF: Recto vaginal fistula
UNFPA: United nations population fund agency
UNFPA: United nations population fund agency
UTI: Urinary tract infection
VUR: Vesicoureteral reflux
VVF: Vesico vaginal fistula
WHO: World health organization
